# Morphological and molecular evolution of hadal amphipod’s eggs provides insights into embryogenesis under high hydrostatic pressure

**DOI:** 10.3389/fcell.2022.987409

**Published:** 2022-09-12

**Authors:** Wenhao Li, Faxiang Wang, Shouwen Jiang, Binbin Pan, Qi Liu, Qianghua Xu

**Affiliations:** ^1^ Key Laboratory of Sustainable Exploitation of Oceanic Fisheries Resources, Ministry of Education, College of Marine Sciences, Shanghai Ocean University, Shanghai, China; ^2^ Shanghai Engineering Research Center of Hadal Science and Technology, College of Marine Sciences, Shanghai Ocean University, Shanghai, China; ^3^ Key Laboratory of Aquaculture Resources and Utilization, Ministry of Education, College of Fisheries and Life Sciences, Shanghai Ocean University, Shanghai, China; ^4^ National Distant-water Fisheries Engineering Research Center, Shanghai Ocean University, Shanghai, China

**Keywords:** hadal trench, amphipod, eggs, high hydrostatic pressure, molecular evolution

## Abstract

Hadal zones are unique habitats characterized by high hydrostatic pressure (HHP) and scarce food supplies. The ability of eggs of species dwelling in hadal zones to develop into normal embryo under high hydrostatic pressure is an important evolutionary and developmental trait. However, the mechanisms underlying the development of eggs of hadal-dwelling species remain unknown due to the difficulty of sampling ovigerous females. Here, morphological and transcriptome analyses of eggs of the “supergiant” amphipod *Alicella gigantea* collected from the New Britain Trench were conducted. The morphology of *A*. *gigantea* eggs, including size, was assessed and the ultrastructure of the eggshell was investigated by scanning electron microscopy. Transcriptome sequencing and molecular adaptive evolution analysis of *A*. *gigantea* eggs showed that, as compared with shallow-water Gammarus species, genes exhibiting accelerated evolution and the positively selected genes were mostly related to pathways associated with “mitosis” and “chitin-based embryonic cuticle biosynthetic process”, suggesting that “normal mitosis maintenance” and “cuticle development and protection” are the two main adaptation strategies for survival of eggs in hadal environments. In addition, the concentration of trimethylamine oxide (TMAO), an important osmotic regulator, was significantly higher in the eggs of hadal amphipods as compared to those of shallow-water species, which might promote the eggs’ adaptation abilities. Morphological identification, evolutionary analysis, and the trimethylamine oxide concentration of *A. gigantea* eggs will facilitate a comprehensive overview of the piezophilic adaptation of embryos in hadal environments and provide a strategy to analyze embryogenesis under high hydrostatic pressure.

## Introduction

Embryogenesis is a highly vulnerable developmental stage that effects the entire life cycle of the organism (Scholz et al., 2008) and ensures production of the target phenotype (Arambourou et al., 2017). Zygote-specific protein-coding genes are expressed during embryogenesis and early organogenesis. Embryogenesis is a complex biological process that integrates mitosis, epiboly, and morphogenesis, including actin organization, microtubule assembly, DNA repair, and cell division (Ishii et al., 2004; Lan et al., 2018). During embryogenesis, abiotic stressors, such as temperature, oxygen concentration, environmental chemical compounds, and high hydrostatic pressure (HHP), can induce failure of embryonic development (Young et al., 1997; Villalobos et al., 2006; Mestre et al., 2008). Embryogenesis is also influenced by various endogenous and exogenous factors that determine the distribution and diversification of the species (Scholz et al., 2008; Ángeles-González et al., 2020).

As the most successful group of animals on the planet, arthropods account for the highest biomass and species diversity (Bar-On et al., 2018). Amphipods are among the most common arthropods in aquatic environments, particularly in hadal zones with the depths exceeding 8,000 m (Blankenship et al., 2006). The hadal zone (depth, 6,000–11000 m) is characterized by low temperatures, lack of light, limited food supply, and HHP (Jamieson et al., 2010). As the most defining environmental factor, HHP increases by 1 atm (or about 0.101 MPa) for every 10 m in water depth (Yancey et al., 2014; Yancey, 2020). Generally, HHP can break protein structures, cause DNA damage, and reduce cell membrane fluidity (Brooks, 2014; Lan et al., 2018). To survive in a hadal zone, chemical chaperones, especially organic osmolytes, facilitate adaptation to HHP. For example, trimethylamine oxide (TMAO) is an important osmotic regulator produced by oxidation of trimethylamine (TMA) via flavin-dependent monooxygenases (FMOs) (Canyelles et al., 2018). The concentration of TMAO is reported to gradually increase with depth, suggesting an important function for organisms occupying hadal zones (Yancey et al., 2014; Qin et al., 2021; Liu et al., 2022). The ability of amphipods to thrive in such extreme habitats and embryogenesis of hadal-dwelling amphipods have attracted much interest.

HHP, a dominant feature of hadal zones, is also reported to delay embryonic development (Mestre et al., 2008), increase the risk of malformations (Mestre et al., 2008), and induce chromosome doubling (Zhu et al., 2017) and aberrations (Glover et al., 2020). Previous studies have reported HHP-induced mitogynogenesis of cultured fish species (Yamamoto, 1999; Lin et al., 2016; Zhu et al., 2017; Glover et al., 2020). However, the pressure tolerance of eggs of other aquatic species remains unknown. The development of the amphipod embryo occurs via ectogenesis, which is defined as growth of an organism in an environment outside of the body. The amphipod’ eggs developing normally in hadal zones under HHP is an important evolutionary developmental trait.

However, investigations of embryonic development of hadal amphipods are limited due to low fecundity (Ritchie et al., 2017), small brood size (Grassle & Sanders, 1973), and fasting during gestation (Perrone et al., 2002; Blankenship-Williams & Levin, 2009). In 2001, a hadal lysianassoid female (*Uristes chastaini*) that was collected with a baited trap while brooding allowed for the first recording of a “live egg” (Blankenship et al., 2006; Blankenship-Williams & Levin, 2009). In 2018, a tiny brood of hatching *Alicella gigantea* (Crustacea, Amphipoda, Lysianassoidea) was collected from the New Britain Trench (7.02S, 149.16E) at a depth of 8,824 m with the use of an autonomous deep-ocean lander. Detailed information about this lander vehicle and the sample collection process is described by Chan et al. (2020; 2022; 2021). To the best of our knowledge, this was the first collection of *A. gigantea* eggs from a hadal zone.

*A. gigantea* is a common inhabitant of hadal zones. In spite of the limited food supply in hadal zones, this species exhibits incredible gigantism, with the adult body length reaching 240–340 mm (Harrison et al., 1983; Barnard & Ingram, 1986; Jamieson et al., 2013). Numerous studies of this “supergiant” amphipod have been conducted, including a mitochondrial genome study of the phylogeny and adaptation mechanisms (Li et al., 2019) and a trace element studies suggested the use of amphipods as potential indicators of trace element bioavailability in hadal zones (Zhu et al., 2022). Transcriptome and evolutionary analyses conducted by Li et al. (2021) identified seven positively selected genes (PSGs) of the “supergiant” *A*. *gigantea* involved in inositol phosphate metabolism, insulin signaling, and glycogenesis signaling, which are ultimately related to growth and proliferation. However, the disjunctive geographical distribution (Jamieson et al., 2013; Ritchie et al., 2015), low observation frequency (Jamieson et al., 2013), and limited genomic resources have hindered elucidation of the adaptation of embryos to the environmental extremes of hadal zones (Jamieson et al., 2013).

Owing to the gigantic genome size of 34.79 pg (34.02 Gb) (Ritchie et al., 2017), expected repeat-rich genomic regions or genome duplication (Ritchie et al., 2017; Wang et al., 2021), assembly of the *A. gigantea* genome remains challenging. Therefore, transcript assembly of the *A. gigantea* egg is an effective option for genome-wide comparative studies of the molecular evolution of embryogenesis of hadal amphipods. Here, the transcriptome of the *A. gigantea* egg was sequenced. Further analysis of the morphology, positive selection, gene evolution rate, and concentration determinations of the TAMO of the *A. gigantea* eggs will facilitate a comprehensive overview of the piezophilic adaptation of embryos in hadal zones and provide a system to study embryogenesis under HHP.

## Materials and methods

### Sample collection and species identification

During an expedition of the New Britain Trench (8,824 m, 7.02◦S, 149.16◦E) in the West Pacific Ocean conducted in 2016, our group collected 10 *A. gigantea* individuals with attached eggs with a full ocean depth lander vehicle launched from the “Zhang Jian” research vessel that were immediately stored at −80 °C immediately. Detailed information about the lander vehicle and sampling process is described by Chan et al. (2020; 2022; 2021) and Li et al. (2021). The specimens were stored in bubble chamber full of dry ice and delivered to Shanghai Ocean University.

Post-spawning eggs adhered to the ventral brood pouch of the brooding female amphipods were carefully separated. The species of the eggs and females were identified based on conventional morphological traits and mitochondrial molecular markers. The *A. gigantea* specimens were identified by mitochondrial cytochrome oxidase subunit Ⅰ (CO I) and 16S rRNA gene markers, which were described by Chan et al. (2020). The COⅠ was amplified using specific primers: COI-F: 5′-GGT​CAA​CAA​ATC​ATA​AAG​ATA​TTG​G-3′ and COI-R: 5′-TAA​ACT​TCA​GGG​TGA​CCA​AAA​AAT​CA-3′. The 16S rRNA gene was amplified using specific primers 16SF: 5′-GAC​GAC​AAG​ACC​CTA​AAA​GC-3′ and 16SR: 5′-CGC TGT​TAT​CCC​TAA​AGT​A-3′.

### Egg measurement and scanning electron microscopy

Eggs were separated from the female amphipods and measured under a ZEISS SteREO Discovery. V20 stereo microscope (Carl Zeiss AG, Oberkochen, Germany). Since the eggs of *A*. *gigantea* are oval, measurements were made along the major and minor axes. All measurements were repeated at least three times and the mean values were calculated.

The *A*. *gigantea* eggs were imaged with a scanning electron microscope. Briefly, the frozen eggs were fixed in 2.5% cold glutaraldehyde solution (4°C) for at least 12 h, as described previously (Tommasini et al., 1989), then dehydrated with a graded series of pre-cooled ethanol (30%, 50%, 70%, 80%, and 90%) for 20 min at 4°C at each concentration. Afterward, the eggs were stored at room temperature and dehydrated with 100% ethanol for 20 min, which was repeated five times. To prevent collapse of the eggshell, the dehydrated eggs were dried with the critical point drying method using a Leica EM CPD300 critical point dryer (Leica Microsystems GmbH, Wetzlar, Germany). The eggs were subsequently coated with gold film using a sputter coater (E-1045, Hitachi High-Tech Science Corporation, Tokyo, Japan) and observed with a scanning electron microscope (SU8010; Hitachi High-Tech Science Corporation).

### RNA extraction, transcriptome sequencing and assembly, and gene functional annotation

Total RNA was extracted from the eggs using TRIzol™ Reagent (Thermo Fisher Scientific, Waltham, MA, United States) in accordance with the manufacturer’s instructions. In this study, three biological replicates of each individual were sequenced. A cDNA library was constructed using a VAHTS Universal V6 RNA-seq Library Prep Kit for Illumina (Vazyme Biotech Co., Ltd., Nanjing, China) and paired-end sequenced using a NovaSeq 6,000 System (Illumina, Inc., San Diego, CA, United States) with a read length of 150 bp in accordance with the manufacturer’s instructions.

To obtain high-confidence assembled transcripts, low-quality bases and adapter sequences were trimmed from the raw sequencing data using the Trimmomatic tool (v0.33; http://www.usadellab.org/cms/index.php?page=trimmomatic) with the following parameters: HEADCROP, 3; AVGQUAL, 30; TRAILING, 20; and MINLEN, 36. *De novo* assembly of the resulting clean reads was performed with Trinity software (v2.4.0; https://github.com/trinityrnaseq/trinityrnaseq/wiki) generated at k-mer = 25 (Haas et al., 2013). The assembled transcripts (contig cut-off > 300 bp) were used for subsequent analysis. The longest isoforms of the genes were selected as unigenes by a custom Perl script to eliminate redundant isoforms. TransDecoder software (https://github.com/TransDecoder/TransDecoder/releases) was used to predict the open reading frames of the non-redundant transcripts. The DIAMOND BLASTX sequence aligner (https://github.com/bbuchfink/diamond) was used to annotate the assembled transcripts of *A. gigantea* eggs in the National Center for Biotechnology Information (NCBI) non-redundant (NR) database (https://www.ncbi.nlm.nih.gov/refseq/about/nonredundantproteins/) with default parameters (e value = 1e-10). Only the best hit was maintained. All contaminated reads were removed and only transcripts identified as Arthropoda taxa were annotated with InterProScan (https://www.ebi.ac.uk/interpro/), Swiss-Prot (https://www.expasy.org/resources/uniprotkb-swiss-prot), NR, KOG (https://mycocosm.jgi.doe.gov/help/kogbrowser.jsf), CDD (http://www.ncbi.nlm.nih.gov/Structure/cdd/cdd.shtml), eggNOG (http://eggnog5.embl.de/#/app/home), Kaas (https://www.genome.jp/kegg/kaas/), and blast2go (v2.5; https://www.blast2go.com/). The gene ontology (GO) annotation results were combined with the results obtained with InterProScan, eggNOG, and blast2go. The Kyoto Encyclopedia of Genes and Genomes (KEGG) annotation results were integrated with the results obtained with Kaas and eggNOG.

### Phylogenetic analysis

For construction of a phylogenetic tree, the embryo transcriptome or genome data of 11 Arthropoda species were obtained from multiple sources. The embryo transcriptome data of *Gammarus chevreuxi* (ERR1097462–ERR1097470) were downloaded from the Sequence Read Archive (https://www.ncbi.nlm.nih.gov/sra), cleaned, and assembled using the same methodology as for the *A. gigantea* embryo. In addition, eight genome sequences and gene annotation files (i.e., *Hyalella azteca*) (Poynton et al., 2018), *Penaeus vannamei* (Zhang et al., 2019), *Armadillidium nasatum*, *Armadillidium vulgare*, *Cryptotermes secundus*, *Daphnia pulex* (Colbourne et al., 2011), *Eurytemora affinis*, and *Trinorchestia longiramus*) were download from the NCBI website (https://www.ncbi.nlm.nih.gov/genome/), while the genome sequence of *Parhyale hawaiensis* (Kao et al., 2016) was download from the Janelia Research Campus website (https://research.janelia.org/Pavlopoulos/). Orthologous protein sequences of each gene were grouped using the OrthoMcl genome-scale algorithm (v2.0.9; https://orthomcl.org/orthomcl/app). In total, 610 groups of single-copy orthologs of 11 species were identified with the use of a manual Perl script. MAFFT multiple sequence alignment software (v7.407; https://mafft.cbrc.jp/alignment/software/) was used to align the sequences, which were concentrated into a supergene with a length of 778,175 aa. Gblocks software (0.91b; http://phylogeny.lirmm.fr/phylo_cgi/one_task.cgi?task_type=gblocks) was used to identify the conserved regions. Finally, 80,824 positions (10%) of 1,742 selected blocks were retained. The topology of the phylogenetic tree was inferred with RaxML software (v8.1.24; https://cme.h-its.org/exelixis/web/software/raxml/) using the best model LG + I + G + F in accordance with the Akaike information criterion and Bayesian information criterion, as determined with ProtTest software (v3.4; https://github.com/ddarriba/prottest3) with *C. secundus* as an outgroup (bootstrap value, 1,000). The graphical viewer Figtree (v1.4.4; http://tree.bio.ed.ac.uk/software/figtree/) was used to visualize the cladogram tree.

### Detection of genes exhibiting accelerated evolution and positively selected genes

Single-copy orthologues were selected among *A. gigantea*, *G. chevreuxi*, *P. hawaiensis*, *T. longiramus*, and *H. azteca* using OrthoMcl software. Considering the distant divergence time, other species were not searched for single-copy orthologues. PRANK software (https://www.ebi.ac.uk/research/goldman/software/prank/software) was used to align the codon sequences based on a codon substitution matrix. The conserved region was obtained using Gblocks software (0.91b) with the parameter “−t = *c*”. Signatures of genes exhibiting accelerated evolution and PSGs along a specific branch were individually detected by branch or branch-site models implemented in the CodeML program of the PAML (phylogenetic analysis by maximum likelihood) package (version 4.9 g) (Yang, 2007). In order to identify genes exhibiting accelerated evolution and PSGs in hadal amphipods, the *A. gigantea* lineage was set as the “foreground” and other shallow-water amphipods as the “background”. The nonsynonymous-synonymous substitution rate ratio dN/dS (*ω*) was used to detect the selective pressure in whole gene level and with codon frequencies calculated average nucleotide frequencies at the three codon positions (*F*3*x*4). Then, Bayes Empirical Bayes (BEB) analysis was used to identify amino acid sites under positive selection. The genes with *p*-value of the likelihood ratio test less than 0.05 were defined as PSGs. GO and KEGG enrichment analyses were implemented with a hypergeometric test using a custom *Python* script. Plotting and statistical analysis were performed with R software (https://www.r-project.org/) and in-house *Python* scripts. The GOChord plot, in which the genes are linked via ribbons to their assigned terms, was generated using GOplot R package (Walter et al., 2015). Orthologous genes of *A. gigantea* and *D. melanogaster* were detected based on reciprocal-best-BLAST-hits (RBH). Protein–protein interaction networks (PPIs) were constructed by STRINIG database (https://cn.string-db.org/; Organisms: *Drosophila melanogaster*). The visualization of PPI networks was implemented using igraph R package (Csardi and Nepusz, 2006).

### Determination of trimethylamine oxide and trimethylamine concentrations in eggs

The concentrations of TMAO and TMA in the eggs of *A. gigantea*, *Cherax quadricarinatus*, *Macrobrachium rosenbergii*, and *Procambarus clarkii* were determined. Measurements of each egg were repeated nine times. Briefly, 20 mg of each egg were added to 1,200 μL of a 4:1 solution of CH_3_OH and water in a centrifuge tube. Then, the eggs were crushed with a high-throughput tissue crusher. Homogenization of the egg tissue revealed that the *A. gigantea* eggshell was quite flexible. The egg suspension was ultrasonicated three times in an ice bath, then chilled to −20°C for 20 min and centrifuged at 13,000 rpm and 4°C for 10 min. The supernatant was collected, diluted by 20 fold, and 400-ml aliquots were stored at 4°C until analysis of the TMAO and TMA concentrations by ultra-performance liquid chromatography–tandem mass spectrometry (Waters Corporation, Milford, MA, United States), as described by Liu et al. (2022).

## Results

### Morphology of *A*. *gigantea* eggs

**FIGURE 1:**
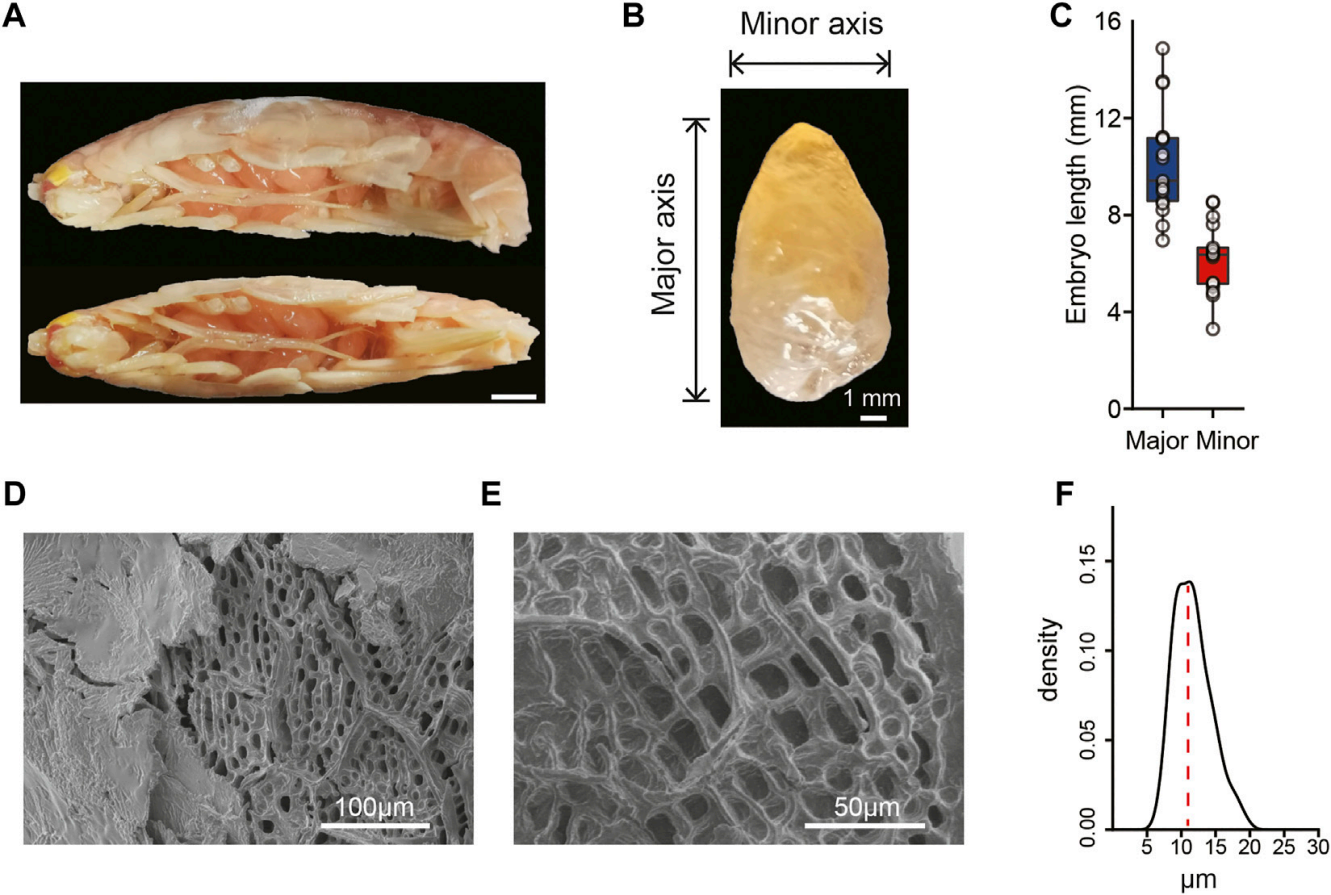
Morphological characteristics of the hadal *A. gigantea*’s eggs. **(A)**. Representative image of *A. gigantea* with eggs (lateral view and vertical view). Scale bar = 10 mm; **(B)**. The image of one *A. gigantea* egg. Horizontal and vertical line segments indicate the minor axis and the major axis, respectively. Scale bar = 1 mm; **(C)**. The length of the minor and the major axis of the measured eggs. **(D)** and **(E)**. The ultrastructure of eggshell by scanning electron microscopy (SEM). Scale bar = 100 μm in D and scale bar = 50 μm in E; **(F)**. The density of the hole diameter in the endochorion of the *A. gigantea*’s eggs.

The post-spawning, but not the free-spawning, “supergiant” hadal amphipods bearing eggs (Figure 1A) were classified as *A. gigantea* based on external morphological traits (Barnard & Ingram, 1986). The eggs collected from the ventral pouch were identified as *A. gigantea* based on conventional morphological traits and mitochondrial molecular markers. Due to the amphipoda egg-collecting behavior (Sheader, 1996) and frozen conditions, the developmental stages of the collected eggs could not be accurately determined. Since no rudimentary embryo was detected microscopically, it was concluded that the sampled eggs were in the early embryogenesis.

*A. gigantea* eggs are oval (Figures 1A,B) with the minor axis ranging from 3.30 to 8.54 mm and major axis ranging from 6.95 to 14.88 mm (Figure 1C). *A. gigantea* eggs can also be considered “supergiant” as compared with the eggs of other amphipod species (Serra, 2003). Scanning electron microscopy revealed that the eggshell of *A. gigantea* was composed of a fibrillar exochorion (Figure 1D) and a porous endochorion layer (Figures 1D,E), similar to the eggshell structure of the fruit flies *Ceratitis capitata* (Margaritis, 1985) and *Rhagoletis cerasi* (Mouzaki & Margaritis, 1991). The mean diameter of the pores of the endochorion was >10 μm, with the largest diameter up to 20 μm (Figure 1F), similar to the perforations of the alveolar layer of the eggshell of *Triops cancriformis,* a crustacean surviving in temporary freshwater pools (Tommasini et al., 1989), but much larger than that of three species of neotropical *Eulimnadia* (Crustacea: Branchiopoda: Spinicaudata) (Rabet et al., 2012).

### Sequencing, assembly statistics, and gene annotation

The transcriptome sequencing results of the *A. gigantea* eggs are summarized in Supplementary Table S1. Sequencing with the Illumina platform returned 179.4 M reads and 26.9 GB of raw data. After filtering, there were 168.9 M (94.15%) clean reads and 24.8 GB of clean data. The trinity-based *de novo* transcriptome assembly consisted of 184,706 contigs. The longest isoform was extracted as a unigene, which consisted of 121,238 contigs. The contig N50 consisted of 628 bp with a total length (nt) of 73.69 M and GC content of 40.30%. Among these, 11,403 contigs were matched with GO annotations. The transcripts with “best-hit” homologs in the NCBI NR database were mostly mapped to Malacostraca, the largest of the six classes of crustaceans. Among these transcripts, 23,273 were mapped to *H. azteca* (Amphipoda), 3,999 to *Penaeus vannamei* (Decapoda), 643 to *Armadillidium vulgare* (Isopoda) and 166 to *C. secundus* (Blattodea).

### Phylogenetic relationships

**FIGURE 2:**
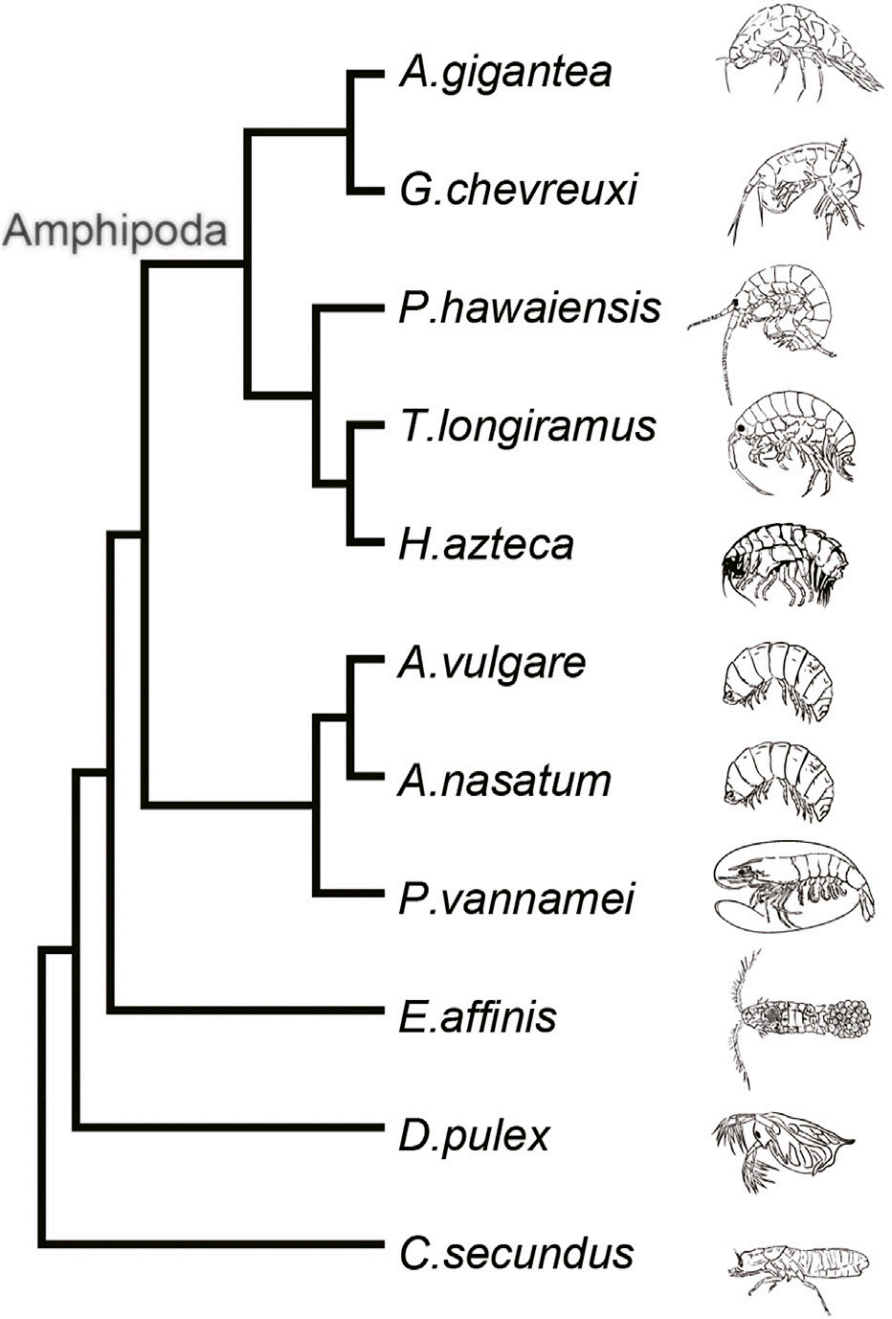
Phylogenetic tree of the 11 arthropods.

A detailed phylogenetic tree constructed from published embryo transcriptomes and whole genome data combined with the transcriptome data of the *A. gigantea* eggs clearly showed that *G. chevreuxi*, a shallow-water amphipod, was most closely related to *A*. *gigantea*, followed by *P. hawaiensis*, *T. longiramus*, and *H. azteca* (Figure 2). All four of these species are members of the order Amphipoda and, thus, used as reference for subsequent analysis.

### Genes exhibiting accelerated evolution and positively selected genes of the hadal lineage

**FIGURE 3:**
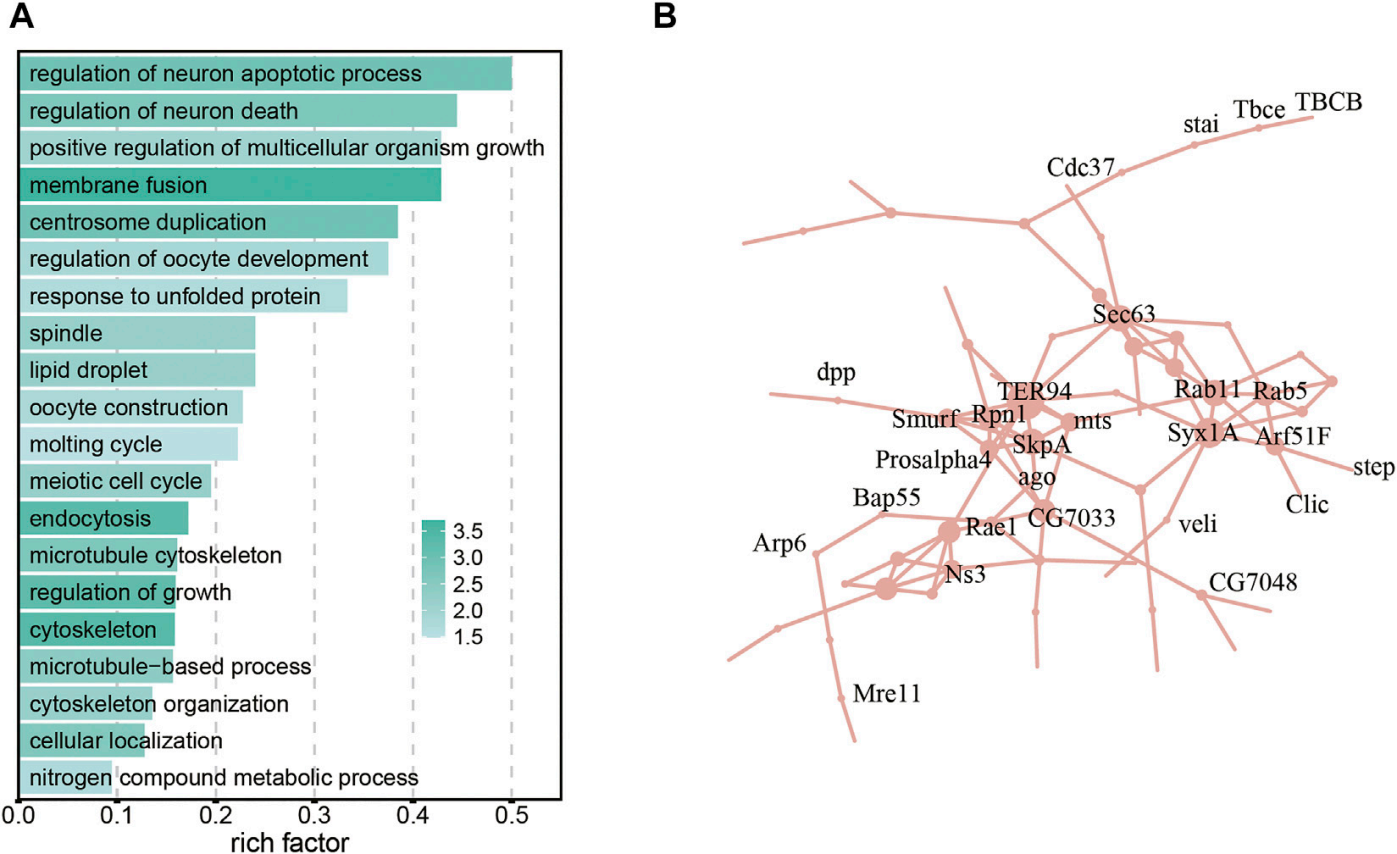
Accelerated evolution of the embryogenesis genes in the eggs of *A. gigantea.* **(A)**. The bar plot shows the GO enrichment results of the accelerated evolution genes; **(B)**. A network of the accelerated evolution genes in the *A. gigantea’s* eggs.

Genetic mutations that conferred an advantage to embryogenesis in the hadal environment were maintained throughout the evolutionary process. Rapidly evolving genes and PSGs were identified from 5-way orthologs genes of *A. gigantea* and shallow-water arthropods, respectively. The lineage of *A. gigantea* was set as the “foreground” phylogeny, and other nodes as the “background” phylogeny. In total, 161 genes exhibiting accelerated evolution were identified in the eggs of hadal amphipods’ (Supplementary Table S2). Enriched pathways associated with genes exhibiting accelerated evolution of *A. gigantea* eggs are shown in Figure 3A. Genes exhibiting accelerated evolution were mainly related to the terms “regulation of neuron apoptotic process”, “regulation of neuron death”, “oocyte construction”, “regulation of oocyte development”, “centrosome duplication”, “spindle”, “microtubule-based process”, “microtubule cytoskeleton”, and “cytoskeleton” (Figure 3A). Then, a network of genes exhibiting accelerated evolution was generated, which included genes associated with embryonic development, such as *dpp* (Figure 3B).

**FIGURE 4:**
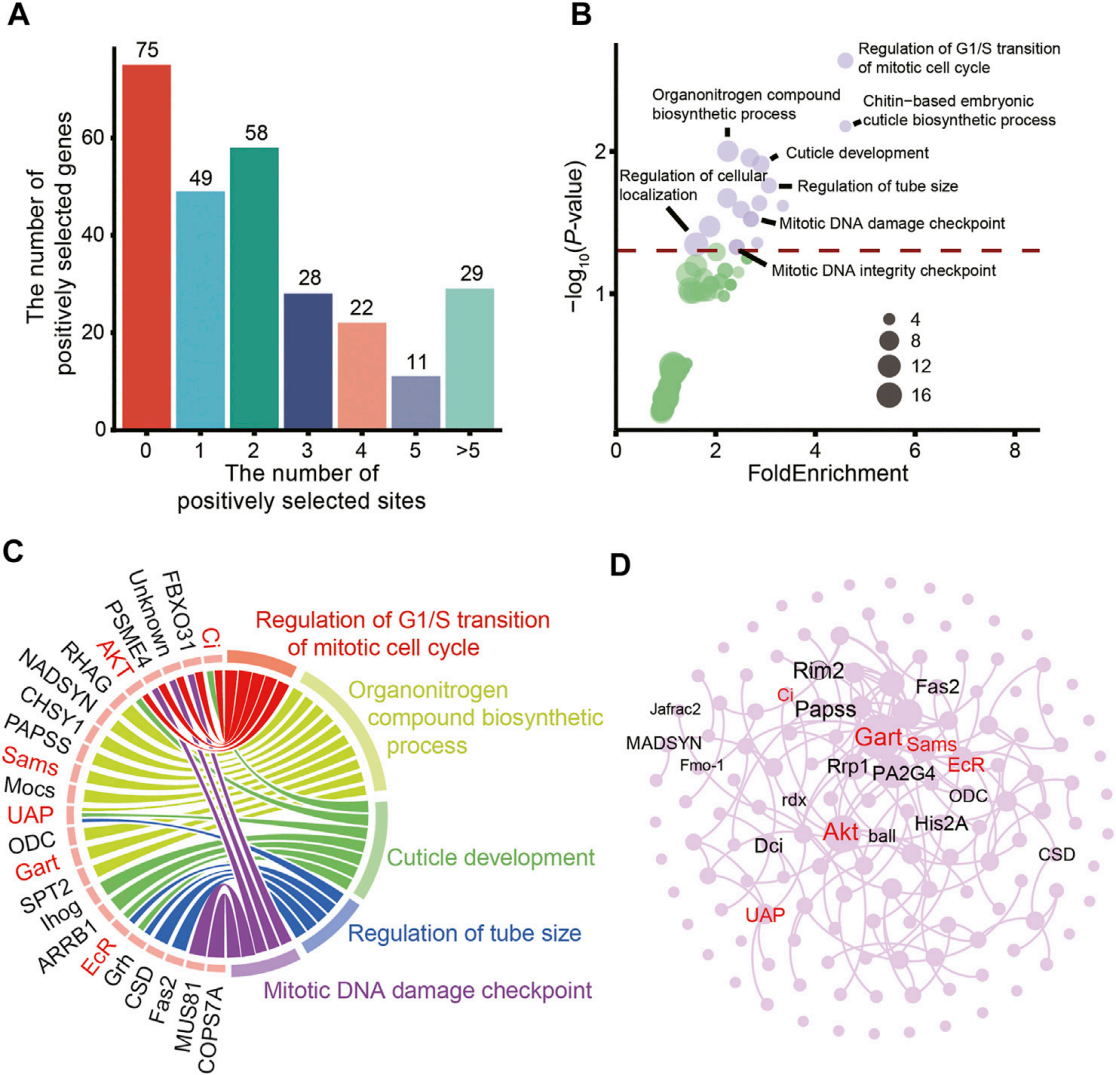
Positively selected genes expressed in the eggs of *A. gigantea*. **(A)**. The distribution of PSGs by positively selected site number; **(B)**. Bubble plot for the enriched KEGG terms; **(C)**. Chord diagram of GO terms and related genes; **(D)**. A network of positively selected genes in the *A. gigantea*’s eggs.

Overall, 272 PSGs were identified in the eggs of hadal amphipods (Supplementary Table S3). Most of the PSGs (182/272) possessed zero, one or two positively selected sites (Figure 4A). The distribution of the positively selected sites of the PSGs of amphipods in extreme hadal environments may provide a reference for subsequent studies of biological adaptability. Moreover, GO analysis showed that most of the PSGs were associated with significantly enriched pathways (*p* < 0.05) involved in cuticle development, DNA repair, cell division, and microtubule regulation (Figures 4B,C). These results demonstrate that HHP in the hadal environment could damage DNA, disintegrate microtubules, and disrupt cell division, suggesting that *A. gigantea* eggs have adapted to HHP in these aspects.

The over-represented GO terms associated with the PSGs also included “chitin-based embryonic cuticle biosynthetic process” and “cuticle development” (Figures 4B,C). The cuticle covering the entire surface of *A*. *gigantea* has multiple functions, such as forming a rigid exoskeleton for muscle attachment, temporary food storage, and formation of a major environmental barrier to prevent physical injury (Vincent and Wegst, 2004). The cuticle is composed of a composite material consisting of crystalline nanofibres embedded in a matrix of proteins, polyphenols, and water, with small amounts of lipids. Seven genes (i.e., *Ci*, *AKT*, *UAP*, *ihog*, *arrb1*, *EcR*, and *grh*) involved in cuticle development were positively selected (Figure 4C). Thus, the relationships among these PSGs were investigated (Figure 4D). The core genes involved with significantly enriched GO terms might interact with surrounding genes. However, further studies are needed to elucidate the specific relationships among these genes and the underlying mechanisms.

### Determinations of trimethylamine oxide concentrations

**FIGURE 5:**
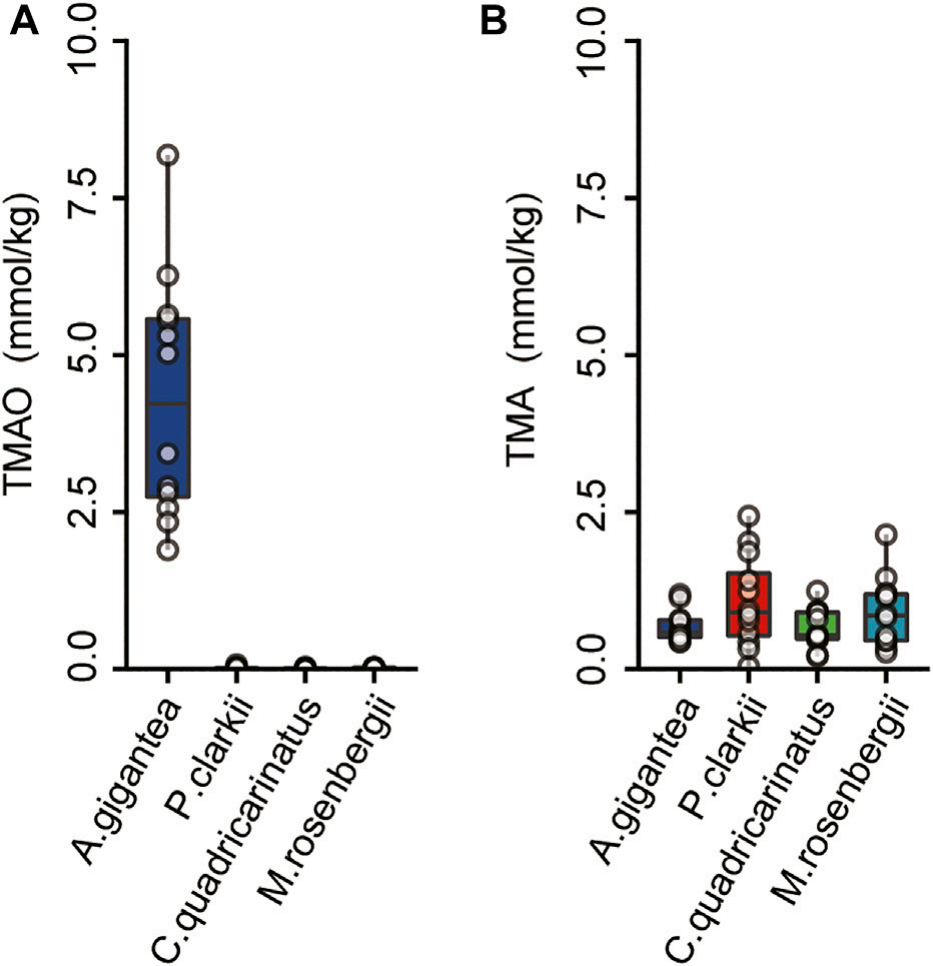
The concentrations of the TMAO and TMA in the eggs of A. gigantea. **(A)** and **(B)**, The content of the TMAO and TMA in the eggs of the A. gigantea, P. clarkii, C. quadricarinatus, and M. rosenbergii.

TMAO plays important roles in the regulation of osmotic pressure in mammals, hadal zone dwellers, and even microbes (Yancey et al., 2014; Qin et al., 2021; Liu et al., 2022). The TMAO content was much higher in the eggs of *A. gigantea* than crustaceans inhabiting shallow waters, while there was no significant difference in TMA contents (Figure 5). These results are similar to those of previous studies that TMAO concentrations are significantly higher in hadal amphipods with similar levels of TMA among different tissues (Liu et al., 2022). TMAO is produced by the oxidation of TMA under the action of the flavin monooxygenases (FMOs) (Canyelles et al., 2018), while TMA is mainly formed by the consumption of carnitine choline by gut microorganisms (Wang et al., 2011). Based on the finding that *A. gigantea* eggs possess relatively high concentrations of TMAO (Figure 5), TMAO might be partially maternally provided, since *A. gigantea* might not generate TMA in the very early stage of embryogenesis.

Moreover, FMO1, one of the main flavin monooxygenase genes to transform TMA into TMAO (Canyelles et al., 2018), was identified as a PSG in *A. gigantea* eggs (Figure 4D and Supplementary Table S3). In the embryo, FMO1 might catalyze maternally acquired TMA to TMAO to enhance survival of *A. gigantea* eggs in hadal environments.

## Discussion

Evolutionary adaptation of adult organisms dwelling in deep-sea environments and hadal zones has been extensively studied in various species, including teleosts (Lan et al., 2018; Wang et al., 2019), bivalve molluscs (Sun et al., 2017), and amphipods (Lan et al., 2017; Li et al., 2021), but rarely in embryos because of the lack of accessibility (Grassle & Sanders, 1973; Perrone et al., 2002; Blankenship-Williams & Levin, 2009; Ritchie et al., 2017). Sampling of gestational amphipods in hadal zones is especially difficult because of limited responses to bait traps and avoided cannibalism, defined as interspecific predation (Fox 1975; Perrone et al., 2002; Blankenship-Williams & Levin, 2009). Some aquatic species are reported to care for eggs in the post-spawning period in deep-sea environments (Seibel et al., 2000; Seibel et al., 2005).

According to previous studies, larval and juvenile amphipods and fish species tend to occupy shallower waters than adult populations (Blankenship et al., 2006; Gerringer, 2019; Weston et al., 2021). Reduced hydrostatic pressure is beneficial to developing instars (Blankenship et al., 2006). However, it is unclear whether brooding amphipods occupy shallower waters in response to pressure or dwell at the same depth as female adults that are not reproductively active (Gerringer, 2019). In this study, brooding amphipod and eggs were collected in the hadal zone, indicating that *A. gigantea* eggs are tolerant to HHP. However, HHP can rupture the egg membrane, resulting in abnormal mass and extrusion of the cytoplasm (Mestre et al., 2008). Therefore, further studies are warranted to explore the mechanisms underlying the adaptation of *A. gigantea* eggs to hadal environments.

### Reproductive traits of hadal species

As compared to shallow-water crustacean species, *A. gigantea* eggs are extremely large, consistent with the characteristics of the eggs of other deep-sea and hadal species (Thorson, 1950; Auel, 2004). Higher energetic investment, such as the production of vitellogenin, is needed to support the development of eggs of deep-sea species (Auel, 2004). However, the larger size of female Arctic deep-sea copepods is not correlated to the larger egg size (Auel, 2004). And no correlation between female size and egg size was found among individuals, such as *Astacus leptodactylus* (Harlioğlu & Türkgülü, 2000). In fact, the egg size of gammarid amphipods is reportedly inversely proportional to temperature during oogenesis. For example, the volume of winter eggs is 60% greater than that of summer eggs (Sheader, 1996). Therefore, the huge egg size of hadal amphipods is possibly an adaptive reproductive trait. The reproductive strategy of K-strategy is to produce few young with high energy content (McLeod et al., 1981). The harsh hadal environment is characterized by fierce competition for resources, less predation pressure, and poor food supply, which results in the K reproductive strategy in *A. gigantea*. As compared to the high reproduction rate of the r reproductive strategy, the K reproductive strategy has evolved as more efficient due to smaller number of eggs, larger egg size, and longer lifespan (Rushton, 1987). In other words, although the overall number is comparatively decreased, egg viability is improved, which may be an adaptation strategy of hadal species to extreme environments. Furthermore, larger eggs might be a ubiquitous characteristic of animals dwelling in harsh environments, such as Antarctic fish surviving in chronic cold (Shandikov & Faleeva, 1992). Therefore, the morphological characteristics of eggs might underlie successful adaptation of amphipods in hadal zones.

### Effects of high hydrostatic pressure on tubulin

HHP has different effects on the assembly of tubulin, actin, myosin II, and cytokeratin in mammalian cells. For example, microtubule is sensitive to high-pressure and excessive hydrostatic pressure can affect the structure of microtubules and cause depolymerization (Zhu et al., 2007; Gao et al., 2018; Yang et al., 2021). HHP is also reported to affect the expression of tubulin (Tahir et al., 1988), regulate development of the cytoskeleton, and induce cytoskeletal deformations (Stramer & Martin, 2005). The sensitivity to high pressure can also change the structure and function of kinesin-microtubule complex. Notably, the GO terms of genes exhibiting accelerated evolution and PSGs were enriched in pathways associated with microtubules and the cytoskeleton (Figures 3, 4), indicating that these genes of *A. gigantea* eggs may have undergone adaptive changes to cope with the HHP in hadal environments.

### Genes exhibiting accelerated evolution related to neuronal cell death

The formation and maintenance of neuronal circuits in the central nervous system is a key process during development (Stephan et al., 2012). Hydrostatic pressure effects on the central nervous system, which might trigger high pressure neurological syndrome (HPNS) (Brauer 1984). HPNS like symptoms such as spasms, convulsions, and tremors have been observed in the shallow-water invertebrates under high pressures, and some genes involved in neuronal DNA damage also showed up-regulations in those shallow-water invertebrates (Morris et al., 2015). Moreover, the neurological impairments, such as convulsions, were also observed in fish under HHP (Sebert 2002). Notably, the roles of adaptive evolution of the nervous system in high hydrostatic pressure deserve better attentions. The neuronal cell death, apoptosis and necrosis can be triggered by excitotoxic damage (Fricker et al., 2018). *In vitro*, pressure would lead to apoptosis by the induction of oxidative stress (Liu et al., 2007) in B35 and PC12 neuronal cell lines (Agar et al., 2000) and in retinal ganglion cells as well (Agar et al., 2006). In this study, genes exhibiting accelerated evolution, including *Psidin/Naa25*, *Naam*, *Torsin* and *TER94*, were significantly enriched in “regulation of neuron apoptotic process” and “regulation of neuron death” (Figure 3A). In *Drosophila*, *Psidin/Naa25* is essential for neuron survival and axon targeting (Stephan et al., 2012). *Naa25*-deficient cells would increase cell apoptosis (Xu et al., 2021). Moreover, the mRNA expression of *Drosophila* nicotinamidase (D-*NAAM*) was found to be up-regulated by the low oxidative stress, which might protect the neuronal cells from oxidative stress-induced cell death (Balan et al., 2008). The inhibition of *Torsin* and overexpression of *TER94* were also reported to induce apoptosis in the cultured cells and the *Drosophila* eyes, respectively (Higashiyama et al., 2002; Chen et al., 2010).

### Evolution of cell-cycle regulators contributes to adaptation to hadal environments

The primary features of embryogenesis are vigorous cell division and proliferation. However, mitosis of eukaryotic cells is inhibited at 30 MPa (Seibel et al., 2000; Gao et al., 2018). In echinoids, high pressure affects the success of early embryonic cleavage (Young et al., 1997). On the other hand, as a mutagen, HHP can induce point mutations (Wang et al., 2015). Evolutionary analysis identified PSGs associated with the terms “mitotic cell cycle”, “supervision of G1 DNA damage checkpoint”, “regulation of G1/S transition”, and “initiation of DNA duplication” (Figure 4). These biological processes are regulated by cyclins and various transcription factors, including *Ci*, *FBX O 31*, *PSME4*, and *AKT* (Figure 4).

As a transcription factor, *Ci* binds to the promoter of *cyclin E* (a G1/S cyclin) to upregulate expression and induce DNA replication and transition to the S phase in cells arrested in the G1 phase (Duman-Scheel et al., 2002). Overexpression of *Ci* is also reported to regulate transcription of *cyclin D* (Duman-Scheel et al., 2002). The dual-function in cell growth and proliferation suggests that *Ci* might be a candidate gene to explain the gigantism of *A. gigantea*.

FBXO31 plays an essential role in G1 arrest and subsequent DNA damage. In mitosis, FBXO31 directly interacts and degrades *cyclin D1* via recognition of the phosphorylated Thr286 residue and promotes ubiquitination of Cyclin D1 (Santra et al., 2009). PSME4A/PA200, a 200-kDa nuclear protein, activates the proteasome by promoting degradation of acetylated histones responding to DNA double-strand breaks (Ustrell et al., 2002; Mandemaker et al., 2018). PSME4A/PA200 is also reported to facilitate DNA repair (Ustrell et al., 2002), replication (MacLellan et al., 2015), and genome stability (Blickwedehl et al., 2008). AKT1 was associated with GO terms associated with pleiotropic effects, including “regulation of cell cycle”, “carbohydrate homeostasis”, and “chitin-based embryonic cuticle biosynthetic process” (Lal et al., 2009). In *Drosophila melanogaster*, AKT phosphorylates *CDKN1B* to control progression of the cell cycle at the G1 stage (Lal et al., 2009).

Therefore, PSGs (e.g., *Ci*, *FBX O 31*, *PSME4*, and *AKT*) expressed in the eggs of *A. gigantea* might regulate mitosis and counteract HHP-induced mutagenesis. So, as an overall evolutionary tendency, cell division is important to the survival of *A. gigantea* eggs in hadal environments.

### Embryonic cuticle development in hadal environments

Multilayered eggshells are produced by follicle cells during oogenesis (Noguerón et al., 2000). In many arthropods, a cuticular egg envelope (blastodermal cuticle in crustaceans; serosal cuticle in insects) is synthesized during early embryogenesis (Lu et al., 2022). During the course of embryogenesis, the blastodermal cuticle is gradually substitutes maternal multilayered eggshell to protect the embryos of arthropods against harsh environmental factors (Vargas et al., 2021).

In the present study, some genes related to embryonic cuticle development, namely cuticle-producing and -organizing factors, were positively selected (Figure 4). Chitin fibers contribute to the stiffness and mechanical resistance of the cuticle (Mrak et al., 2017). UDP-N-acetylhexosamine (UDP-HexNAc) pyrophosphorylase (UAP) is involved in the biosynthesis of chitin, an essential component of the larval cuticle (Stramer & Martin, 2005). Ecdysteroids play an important role in the deposition of chitin in the serosal cuticle. Upregulated expression of ecdysone, the active form of ecdysteroid, triggers the formation of the serosal cuticle (Lagueux et al., 1979; Goltsev et al., 2009).

In addition, the ecdysone receptor (EcR) is also important for development of the embryonic cuticle. EcR is required for maximal expression of the cuticle protein-coding gene *EMWCP10* (Wang et al., 2010). Tissue-autonomous EcR is required for concurrent organ morphogenesis in the *Drosophila* embryo. EcR is a major regulator of tissue development and growth in the marine salmonid ectoparasite *Lepeophtheirus salmonis* (Copepoda, Caligidae).

The transcription factor *grh* is also involved in cuticle formation. *Drosophila grh* regulates epidermal cuticle formation and plays essential roles in wound healing (Mace et al., 2005; Stramer & Martin, 2005). Positive selection of these genes indicates that the embryonic cuticle development of *A. gigantea* is involved in adaptation to hadal environments.

### Trimethylamine oxide in the eggs of hadal amphipods

Proteins tend to lose folding ability under high pressure. Thus, species that dwell in hadal zones need pressure-counteractant to improve protein stability and functional adaptability. TMAO is reportedly important for adaptation of various species to hadal environments (Yancey, 2020). Stress-induced perturbations to the tertiary structure of proteins transcend the protective capacity of TMAO, the heat shock protein could be induced in spiny dogfish shark (Villalobos & Renfro, 2007; MacLellan et al., 2015). As a chemical chaperone, TMAO may be the first line of defense against stress-induced damage and replace functions of other molecular chaperones.

Previous studies have reported that TMAO concentrations are significantly higher in the tissues of hadal species than shallow-water species (Gillett et al., 1997; Samerotte et al., 2007; Liu et al., 2022). Likewise, the results of the present study revealed that TMAO contents were significantly higher in the eggs of hadal amphipods than those of shallow-water crustaceans, while TMA concentrations were similar (Figure 5). TMAO is an important molecule for the survival of hadal species. The high content of TMAO in *A. gigantea* eggs might be important to ensure normal egg development under HHP conditions. FMO1 is highly expressed in the human embryo (Koukouritaki et al., 2002) and may be involved in maternal interactions (Ishida et al., 2007). Thus, the positively selected FMO1 gene and TMAO play an important function in the eggs of amphipods in hadal environments.

### Developmental regulation of amphipod eggs in hadal environments

**FIGURE 6:**
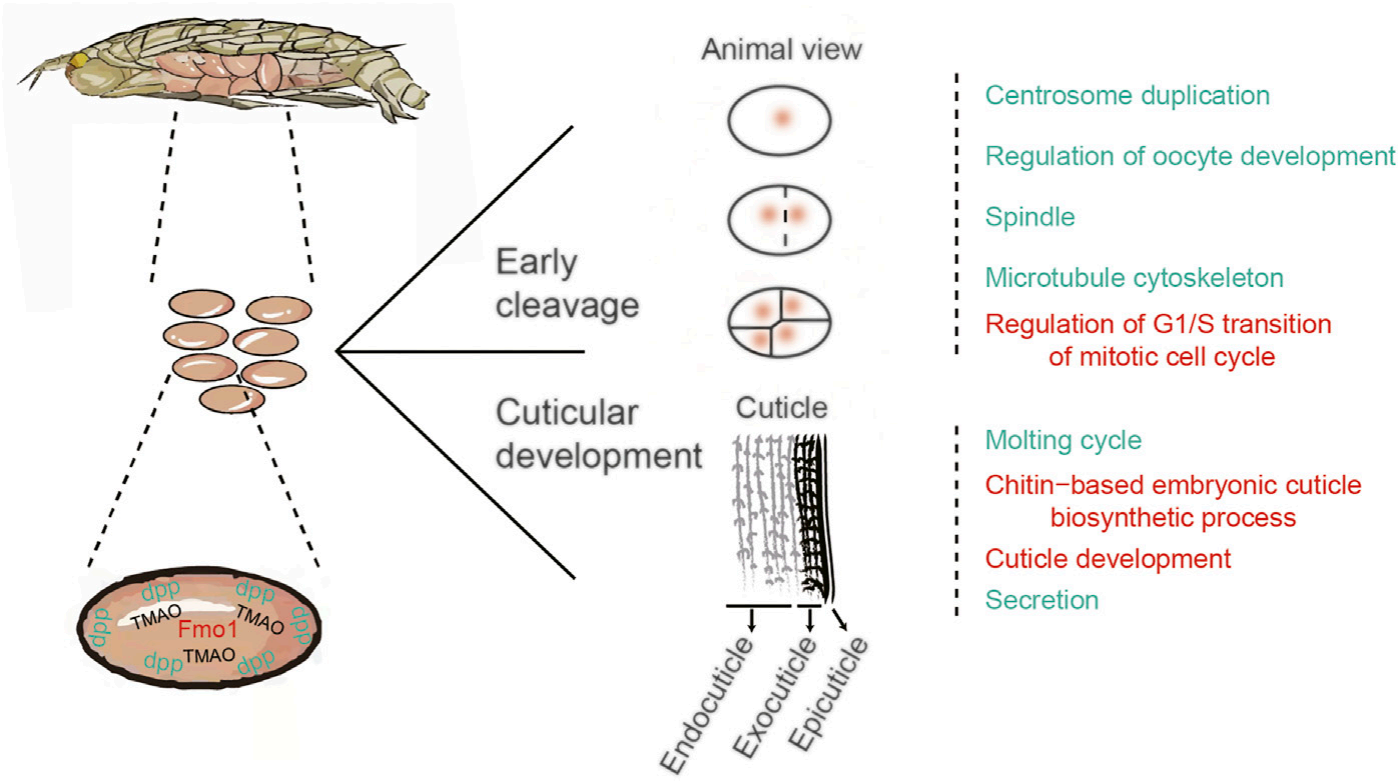
A schematic representation of the adaptive molecular evolution of the A. gigantea embryo.

PSGs combined with genes exhibiting rapid evolution were divided into two groups based on associations with mitosis in the early cleavage stage and cuticle development (Figure 6). Genes involved in the early cleavage stage (i.e., *Ci, Fbxo31*, and *AKT*) are mainly related to the GO terms “centrosome duplication”, “regulation of oocyte development”, “spindle”, “microtubule cytoskeleton”, and “regulation of G1/S transition of mitotic cell cycle” (Figure 6). Positive selection of these genes ensures normal cleavage and development of *A. gigantea* eggs in the early stage.

The PSGs *ihog*, *UAP*, *ECR*, and *GRH* are mainly related to the molting cycle, chitin-based embryonic cuticle biological processes, and cuticle development and secretion. These PSGs might facilitate adaptation of the *A. gigantea* cuticle to hadal environments (Figure 6). In *Drosophila*, *dpp* is important to embryonic development, formation of the dorsal-ventral axis, and establishment of the early embryonic shape (Auman & Chipman, 2017), and might also regulate related genes involved in the formation of the *Drosophila* eggshell and appendages (Osterfield et al., 2017). The rapid evolution of *dpp* indicates that the *A. gigantea* eggshell has undergone adaptive evolution to cope with harsh hadal environments. Meanwhile, the large amount of TMAO in *A. gigantea* eggs is believed to maintain the stability and normal function of proteins involved with egg development.

By comparing the results of the eggs with the adults transcriptome analysis (Li et al., 2021), we found the PSGs obtained from the eggs and the adults were both involved in “cell cycle regulation”. However, in the adults whole body, PSGs were closely related to meiotic or mitotic process, such as “meiotic cell cycle”, “female meiotic nuclear division”, “spindle pole”, “mitotic prometaphase” and “condensed chromosome” (Li et al., 2021). Meiosis is a special type of cell division for gametogenesis in gonad. In the eggs, PSGs were enriched in mitotic process, such as “Mitotic DNA damage checkpoint” and “Regulation of G1/S transition of mitotic cell cycle”. The early embryo is characterized by intensive mitotic cell division. Furthermore, the embryonic development of arthropods has sufficient nutrient supply from yolk components (Silveira et al., 2006). This might be the reason why “response to starvation” was enriched in whole body but not enriched in PSGs in the eggs. Since cuticle formation happens during embryonic development of Arthropoda (Ostrowski et al., 2002), the eggs are excellent specimen to investigate the genetic control of cuticle development. The “cuticle development” and “chitin-based embryonic cuticle biosynthetic process” were enriched in the eggs, which certainly can’t be unfolded by the adults study. Collectively, these findings deepen the current understanding of embryogenetic adaptations of hadal amphipods to HHP and provide valuable genetic resources for adaptation to hadal environments.

## Data Availability

All sequencing data associated with this project were deposited in the National Center for Biotechnology Information (NCBI) Sequence Read Archive database (BioProject Accession Number: PRJNA841239).
